# Neurobiological effects of music-making interventions for older adults: a systematic review

**DOI:** 10.1007/s40520-025-03006-7

**Published:** 2025-04-02

**Authors:** Nicole Espinosa, Zoe Menczel Schrire, Andrew C. McKinnon, Hannes Almgren, Loren Mowszowski, Sharon L. Naismith

**Affiliations:** 1 School of Psychology, Faculty of Science, University of Sydney, Sydney, Australia; 2 Healthy Brain Ageing Program, Brain and Mind Centre, University of Sydney, Camperdown, Sydney, NSW 2050 Australia; 3 Charles Perkins Centre, University of Sydney, Camperdown, Sydney, NSW 2050 Australia; 4 School of Biomedical Engineering, Faculty of Engineering, University of Sydney, Sydney, Australia; 5 School of Psychology, Western Sydney University, Sydney, Australia

**Keywords:** Ageing, Neuroplasticity, Music-intervention, Imaging, MRI, Older adults, Piano, Choral singing, Grey matter

## Abstract

**Background:**

Evidence on the impact of music-making interventions on brain plasticity in older adults is limited.

**Aims:**

To investigate whether music-making interventions in older adults induce neurobiological changes and if such changes relate to cognitive improvements.

**Methods:**

A systematic search was conducted in Medline, PsycINFO, and Scopus. Inclusion criteria targeted randomised controlled trials with older adults (with and without mild cognitive impairment [MCI]), music-making interventions as exposure, and neurobiological measures as the primary outcome.

**Results:**

Six studies (555 cognitively intact older adults) met inclusion criteria—five used piano training, one used choral singing. Three studies had overlapping cohorts, and four had a high risk of bias. One study employed electroencephalography (EEG) to measure frontal and parietal activity, while five used structural MRI to assess cortical, subcortical, and white matter integrity. Methodological heterogeneity limited comparability. Findings in the piano group included increased frontal theta power during an improvisation task, greater grey matter volume in the dorsolateral prefrontal cortex and cerebellum, slower fibre density decline in the fornix and preserved grey matter volume in the right auditory cortex and hippocampus. Only one study reported a positive correlation between neurobiological changes and executive functioning improvements. No studies assessed neurobiological outcomes in MCI.

**Discussion:**

Evidence on music-making interventions and neuroplasticity in older adults remains inconclusive due to limited studies, high risk of bias, and methodological variability. While preliminary findings suggest potential neurobiological changes with music-making interventions, there is insufficient evidence to draw firm conclusions.

**Conclusions:**

High-quality trials are needed to clarify the neurobiological impact of music-making, particularly in MCI populations.

**Supplementary Information:**

The online version contains supplementary material available at 10.1007/s40520-025-03006-7.

## Background

The proportion of older adults in the population is increasing globally [1]. The United Nations predicts that by 2050, older adults will make up approximately 30% of the population in developed nations [2]. The changing demographics of our society pose unique challenges, especially in terms of addressing age-related problems, such as cognitive and physical deterioration. Cognitive decline, in particular, is a growing concern given that it is associated with increased healthcare costs, functional disability, financial burden, and psychiatric symptoms [3–5]. Further, around 15% of older adults worldwide meet criteria for mild cognitive impairment (MCI) [6], widely recognised as a high-risk stage between healthy ageing and dementia, with approximately 45% of individuals converting to dementia within five years [7]. In MCI, significant cognitive decline is evident on neuropsychological testing (typically 1.5 standard deviations (SD) below the premorbid expected level of functioning) while daily functioning remains intact [8]. Notably, evidence suggests that early interventions targeting modifiable risk factors may promote brain plasticity (the brain's ability to reorganise and adapt its structure and functions in response to experiences, learning, or environmental changes) [9] and improve cognition in older adults with cognitive difficulties and may even reduce dementia prevalence [8]. This highlights the critical need to develop interventions that can either prevent or slow cognitive decline in older persons.

Of interest, there is a close coupling between patterns of cognitive decline and neurobiological brain changes across the spectrum of healthy ageing and many neurodegenerative diseases. Alzheimer’s disease (AD) is characterised by the hallmark neuropathological features of both beta-amyloid (Aβ) deposition and tau aggregation [10–14], as well as changes in functional brain connectivity [15]. Positron Emission Tomography (PET) studies have found that 55% and 27% of older adults who meet clinical criteria for MCI have evidence of Aβ deposition and pathological tau, respectively [16]. In addition to AD pathology, individuals with MCI may have concomitant cerebrovascular disease, and many will have other causes of neurodegeneration, such as those linked with synucleinopathies or frontotemporal dementia [7].

Regardless of the pathophysiological underpinning, evidence indicates that the reduction in cognitive functions in the context of ageing is, to some extent, mediated by both structural and functional changes in the brain [17, 18]. For example, changes in memory may be mediated by alterations in the hippocampus, a structure affected early in the course of AD and in MCI [18–21]. There are also reductions in cortical thickness and grey matter volumes, especially in the frontal and parietal lobes [22, 23], as well as the thalamus, putamen, and nucleus accumbens [17, 24–26]. Diffusion-weighted imaging (DWI) studies have suggested that the integrity of white matter in our brains deteriorates as we age. Several DWI studies have reported that the integrity of white matter (WM) (based on diffusion tensors) can mediate the relationship between age and cognitive functions [27–29], suggesting that WM integrity may play a causal role in cognitive decline. Studies examining white matter hyperintensities (WMHs) similarly show that a higher WMH load is associated with more severe cognitive decline, particularly in processing speed and executive functions [30]. WMHs have also been linked to an increased risk of developing vascular dementia and AD [31]. Moreover, several resting-state functional magnetic resonance imaging (MRI) studies have revealed reduced default mode network functional connectivity in MCI patients compared to controls [32, 33] that are, in turn linked to early changes in memory [15]. Electroencephalography (EEG) studies have identified alterations in the theta band and significant power spectrum differences between healthy older adults and those MCI [34, 35].

These imaging studies support the notion that abnormal neurobiological changes may contribute to the cognitive deficits observed in ageing and MCI, highlighting the importance of utilising brain imaging markers for assessing the effect of interventions on brain plasticity in older adults with and without MCI. Importantly, studies have suggested that neuroplasticity can still occur in older adults in relation to interventions [36–38], including musical interventions [39]. Thus, there may be a critical window, preceding dementia, by which primary or secondary prevention treatments can be tested for their ability to promote brain plasticity, and, in turn, help to ameliorate cognitive deterioration and maintain an individual’s quality of life.

While new disease-modifying monoclonal antibody therapies for MCI due to AD and early AD are promising [40, 41], cholinesterase inhibitors are currently the most commonly used symptomatic treatment for older adults with dementia. However, only modest short-term cognitive benefits have been observed in clinical trials [42]. Further, these drugs are not indicated for those with MCI, and they do not target neuroplasticity or brain resilience. In addition, drug-related side effects are a consideration for both drug treatments [40, 41, 43]. International and national regulatory bodies still recommend the use of non-pharmacological interventions as first-line treatments to improve or maintain the cognitive status of older adults at risk for dementia [44–46].

Such non-pharmacological approaches include lifestyle modifications relating to nutrition and exercise, as well as cognitive interventions incorporating drill-practice exercises and/or cognitive strategy training. For example, in people with MCI, meta-analytic studies have shown that cognitive interventions are associated with moderate effect size improvements in verbal memory and nonverbal learning [47, 48], processing speed and visual-spatial skills [49], and have positive effects on everyday life [50], promoting cognitive health and well-being [51, 52]. While cognitive interventions are clearly effective and beneficial, they are not widely available, may not appeal to all, and may not be generalisable or sustainable.

Music-making interventions are a potentially more engaging and enjoyable way to deliver interventions targeting cognition, and can be delivered in the community. Learning a new skill, such as playing an instrument or joining a choir, may even be suggested to older people in order to optimise healthy brain ageing [53, 54]. Not only can such activities be cognitively stimulating, but therapies based on real-life experiences (e.g. music) can facilitate the transfer of learning naturally, as they are more intricate and diverse compared to laboratory training [55]. While many people readily enjoy listening to music, engaging more actively with *making* music is also an accessible, intrinsically motivating, and often aspirational experience. Multiple reviews have evaluated the effect of music-making interventions on cognitive function in older adults at risk for dementia [56–59]. For instance, a recent systematic review and meta-analysis of 19 studies with 1024 MCI or dementia participants revealed small to moderate significant improvements in executive functioning *(Cohen’s d* = 0.27, CI = [0.10;0.44]), episodic memory (*Cohen’s d* = 0.34, CI = [0.08;0.61]) and global cognition (*Cohen’s d* = 0.35, CI = [0.10;0.59]) in association with music-making interventions [57].

Despite these promising results for cognitive effects, to our knowledge, no systematic reviews have been conducted to identify underlying neurobiological changes (e.g. structural, functional, or molecular brain changes) associated with music-making interventions in cognitively intact older adults and older adults with MCI. This is a crucial gap in the literature, as brain imaging alterations have been shown to predate changes in cognition [60, 61] and, therefore, are not only useful for early detection but could also provide early mechanistic insights regarding the impact of interventions on neuroplasticity.

The primary aim of this study was to conduct a systematic review to investigate whether music-making interventions in this population are associated with changes in structural, functional or molecular neuroimaging markers. As a secondary aim, we sought to evaluate if such neurobiological changes are linked to improvements in cognitive performance.

## Methods

### Protocol and registration

This systematic review was conducted according to Preferred Reporting Items for Systematic Reviews and Meta-Analysis (PRISMA) guidelines [62] and was registered with PROSPERO (registration number ID: CRD42023444219).

### Information sources and search terms

Searches of Medline, PsycINFO, and Scopus databases were initially conducted in July 2023 with the last search made on 28th January 2025. In this phase, we consulted a librarian to ensure that our search strategy was satisfactory. The search terms chosen were included in the following Boolean expression: ‘Music intervention’ OR ‘Music training’ OR ‘Music making’ OR ‘Music’ OR ‘Piano’ OR ‘Choir’ OR ‘Singing’ AND ‘Older adults’ OR ‘Ag*ng’ OR ‘Elderly’ OR ‘Mild Cognitive Impairment’, OR ‘Cognitive Decline’ OR ‘preAlzheimer’ OR ‘at-risk’ AND ‘Imaging’ OR ‘Neurobiological’ OR ‘Neuroanatomy’ OR ‘Neuroplasticity’ OR ‘Magnetic Resonance Imaging’ OR ‘Grey matter volumes’ OR ‘electroencephalogram’ OR ‘Cortical Thickness’ OR ‘Bold signal’ OR ‘White matter’. All key search terms were combined, where possible, with indexed terms and medical sub-headings (MeSH) to identify potentially relevant studies. We did not include any limitations on language or publication date. The complete list of search terms can be found in the Supplementary material. We also manually searched through the reference lists of relevant original and review articles that were found during our search, to identify any additional relevant studies.

### Study selection

All studies identified by our search strategy were imported into the Covidence online software platform [63]. The software automatically removed duplicate articles. Two reviewers (NE, ZMS) completed the initial screening review for eligible studies. The reviewers independently screened titles and abstracts to identify studies that potentially met the inclusion criteria. Any discrepancies were resolved by discussions between the two reviewers and consulting a third review author (AM) when needed. The following inclusion criteria were used:

1. *Type of study:* either a published or unpublished randomised controlled trial (RCT).
2. *Population*: older adults over 50 years classified as cognitively intact older adults or older adults with a clinical or research diagnosis of MCI according to accepted criteria (e.g. Petersen [7], Winblad [64], Albert (research criteria) [65]). We excluded studies if participants had received a formal diagnosis of dementia, other neurological conditions (e.g. Parkinson’s or Huntington’s diseases) or psychiatric disorders (e.g. bipolar disorder, schizophrenia).
3. *Comparison:* The control participants needed to be adults who were similar in age to the participants in the intervention group (at the group level). To confirm that the control group did not have any cognitive impairment, the same clinical and cognitive assessments used to diagnose MCI were also have been administered to them.
4. *Intervention:* Music-making interventions were defined as those that utilise instrument playing (e.g. piano) or singing as primary intervention methods to improve or maintain cognitive function in older adults with or without MCI. We excluded studies that used other types of music-related interventions such as music listening, music therapy, single-frequency sound or vibrotactile stimulation.
5. *Outcome*: The primary outcome (neurobiological brain changes) included any neurobiological brain alterations, such as brain structure, function, or molecular brain changes. Measurement of main outcomes needed to be conducted with well-established brain imaging techniques, such as MRI or electroencephalogram.
6. *Original data:* The study needed to report original data and could not be an editorial, commentary, conference abstract, or review.

**Fig. 1:**
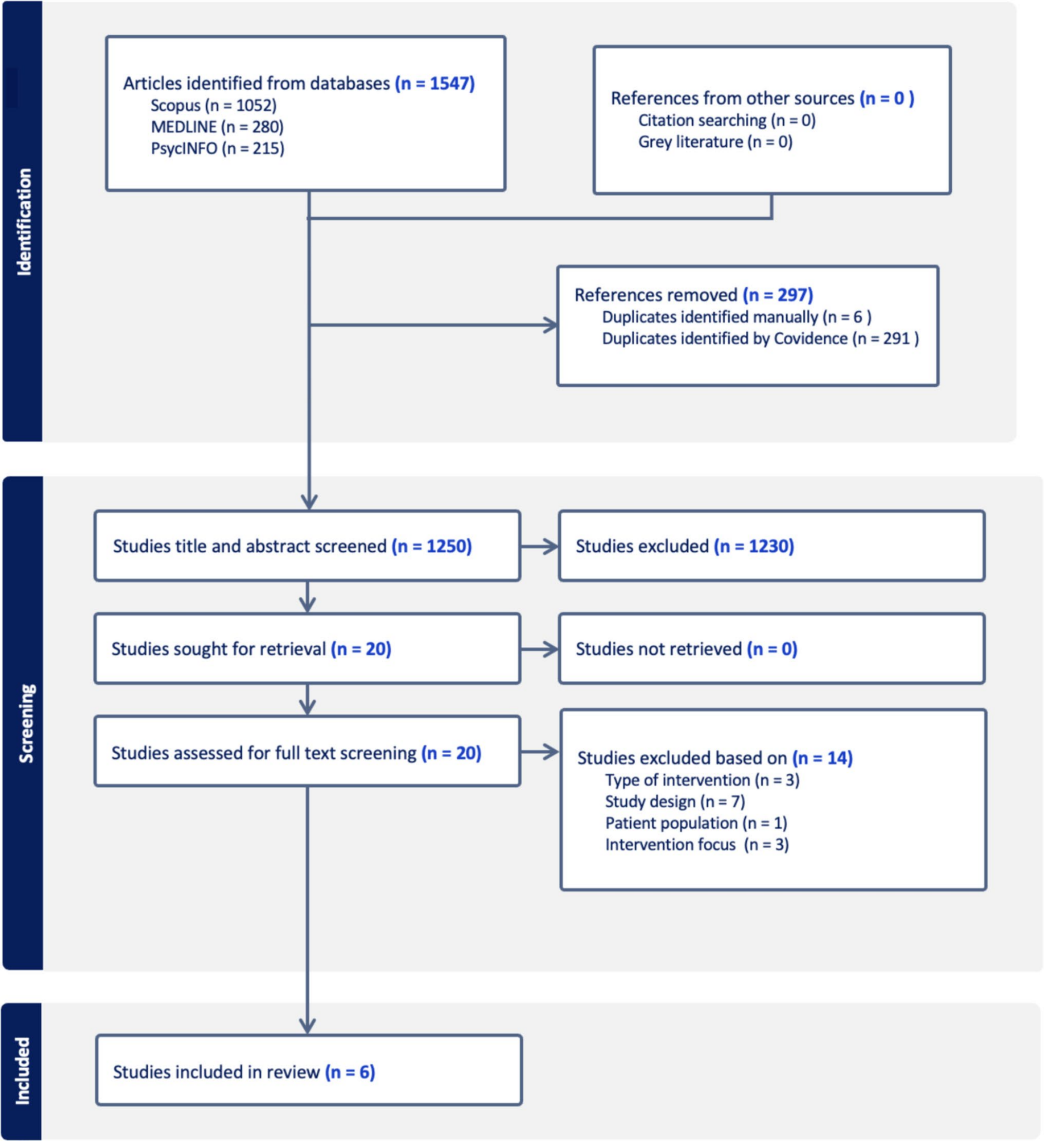
PRISMA flowchart

Then, the full texts of these potentially eligible studies were obtained for further screening by the same two reviewers (NE, ZMS), and disagreements were resolved by consensus with a third author (ACM). The number of included and excluded studies are presented in a flowchart (Fig. 1).

### Data extraction

The identification of relevant information for extraction was determined a priori. Information from each study was extracted into a standardised data extraction table in parallel by two reviewers (NE, ZMS). The extracted data include study design, participants’ cognitive diagnosis, participant demographics, and intervention details. Neuroimaging techniques and analysed brain regions-of-interest (ROIs), as well as cognitive outcomes in each study were also recorded. Due to the heterogeneous nature of the studies, and the variation in study designs and methods, the extraction of meaningful effect sizes for comparison and further quantitative meta-analysis was not possible. Therefore, the results are presented descriptively.

### Risk of bias

The risk of bias was evaluated by a committee of two investigators (NE, ZMS) using the revised Cochrane Risk of Bias Tool (RoB 2.0) for RCTs [66]. The RoB 2.0 consists of the following five domains: randomisation process, deviation from intended interventions, missing data, measurement of the outcome, and selection of reported results. As per guidelines, studies were classified as overall high risk of bias if they had medium risk of bias in multiple domains or a high risk of bias in any domain, using discretion by the authors.

## Results

### Study selection

An initial search yielded 1547 articles from Medline (n = 280), PsycINFO (n = 215), and Scopus (n = 1052). After duplicate removal, there were 1250 articles remaining for title and abstract screening. As shown in Fig. 1, subsequently, the full-texts of 20 articles were checked for eligibility against the inclusion criteria, and a further 14 articles were excluded. Our final sample comprised six full-text articles [67–72]. The primary reason for the exclusion of full-text articles was the ineligible study design (n = 7). This was followed by the type of intervention (n = 3) and the intervention focus, where the target was not to improve or maintain cognitive function (n = 3) (for more details, see Fig. 1). No further eligible articles were found through our manual search of reference lists from relevant reviews or from the included articles.

### Study characteristics

Data extraction was completed for six studies. The characteristics and outcomes of the included studies are summarised in Table 1. In terms of RCT methodology, five studies used a parallel design [67–70], and one used a matched-paired design [71]. It is important to highlight that three [69–71] of the studies included in this review used data from the same participants obtained through the same longitudinal study [73] conducted in Hannover (Germany) and Geneva (Switzerland).

**Table 1:** Characteristics of included randomised controlled trials

First author, (publication year)	Cognitive status (HOA/SCI)	Study design	Baseline characteristics by group			Intervention characteristics			Outcome measures	
			Music -making intervention N, sex, mean age (SD)	Active control N, sex, mean age (SD)	Passive control N, sex, mean age (SD)	Music-making (duration/intensity)	Active control (duration/intensity)	Passive control (duration/intensity)	Modality: Brain regions outcomes^+^	Cognition
Bugos (2024)	HOA	Parallel	20, 10F, 69.65 (4.9)	n/a	20, 13F, 68.2 (4.1)	Jazz piano training (2 weeks; 10 sessions; 3 h each session)	n/a	Passive control (2-weeks)	EEG: Frontal and parietal regions	The Cued Color Word Stroop, PASAT, D-KEFS verbal fluency subtest
Feng (2020)^**^**^	SCI	Parallel	47, 36F, 71 (5.7)	46, 37F, 69.4 (5.3)	n/a	Choral singing (2 years; 60 min, once a week)	Health education program (2 years; 60 min, once a week)	n/a	DWI: Global GM and WM T1: Hippocampus, total brain volume, total GM volume, total WM volume, and total ventricular volume	Composite cognitive test score
Jünemann (2022)*	HOA	Parallel	59, 32F, 69.5 (3.2)	62, 37F, 69.42 (3.8)	n/a	Piano training (6 months; 60 min, once a week, 30 min homework each day)	Music listening/musical culture lessons (6 months; 60 min, once a week, 30 min homework each day)	n/a	DWI: Corpus callosum, fornix, acoustic radiation, corticospinal tract, and arcuate fasciculus	RAVLT delayed recall condition
Marie (2023)*	HOA	Parallel	66, 38F, 69.2 (3.2)	66, 39F, 69.2 (3.8)	n/a	Piano training (6 months; 60 min, 30 min homework each day)	Music listening/ musical culture lessons (6 months; 60 min, 30 min homework each day)	n/a	T1: Whole-brain GM volume, cerebellum, caudate nucleus, Rolandic operculum and primary auditory cortex	Tonal working memory task, and Backward Digit Span
West (2017)	HOA	Parallel	12, 10F, 67.7 (4.3)	8, 4F, 69.3 (5.7)	13, 10F, 66.9 (3.9)	Piano training (6 months; minimum 30 min, 5 days a week)	Video game training (6 months; minimum 30 min, 5 days a week)	Passive control (6 months)	T1: Hippocampus, cerebellum, and dorsolateral prefrontal cortex	MoCA and short-term memory performance
Worschech (2023)*	HOA	Matched-paired	136, 80F, 69.7 (3.6)^1^		n/a	Piano training (12 months; 60 min, 30 min homework each day)	Music listening/ musical culture (12 months; 60 min, 30 min homework each day)	n/a	T1: M1, thalamus and putamen	Digit Symbol test and Digit Span Backward test

*SCI* Subjective cognitive impairment, *HOA* Healthy older adults, *EEG* electroencephalography, *PASAT* Paced Auditory Serial Addition Task, *D-KEFS* Delis-Kaplan Executive Function System, *DWI* Diffusion-weighted imaging, *GM* Grey matter, *WM* White matter, *MoCA* Montreal Cognitive Assessment, *RAVLT* Rey Auditory Verbal Learning Test, *M1* Primary motor cortex, *n/a* non-applicable

*These studies used data from the same longitudinal study conducted in Hannover (Germany) and Geneva (Switzerland)

^1^Total sample size, groups were matched by sex and age

+ All brain region outcomes were pre-specified by a hypothesis-driven approach

^Imaging outcomes were secondary endpoints of this research study

Five of the studies were conducted in healthy adults [68–71], while one included individuals with subjective cognitive decline (SCD) [67]. None of the included studies examined MCI participants. Cognition was the primary outcome in the study by Feng et al. [67] and a co-primary outcome in the studies by Bugos et al. [72] and Worschech et al. [71]. In the remaining studies, cognition was considered a secondary outcome [68–70]. In five studies with healthy older adults, a global cognitive functioning test (e.g. Cognitive Telephone Screening Instrument [69–71] or the Montreal Cognitive Assessment [68, 72]) was utilised to assess cognitive decline. Only one study [67] with individuals with SCD used a comprehensive, standardised neuropsychological test battery.

In terms of intervention design, the most commonly employed intervention to evaluate the impact of music-making on neurobiological changes was piano training [68–72]. Only one study utilised choral singing as an intervention [67]. The five studies that implemented piano training [68–72] explicitly recruited musically naïve participants, whereas the study using choral singing intervention [67] did not specify participants’ prior musical experience. Further, the control groups either received another type of active intervention (i.e. music listening [69–71], health education program [67] or video game [68]), or had no intervention (i.e. passive control) [68, 72].

Neuroimaging was a primary trial outcome in three studies [68–70] and a co-primary outcome in two studies [71, 72], while it was a secondary outcome in the study by Feng et al. [67]. All six studies used a hypothesis-driven approach for their primary analysis and had pre-specified regions of interest. Examination of neuroimaging modalities across the studies revealed that five studies utilised structural MRI methods [67–71], whereas only one study employed EEG [72] to assess the neurobiological effects of the music-making intervention. In Bugos et al. [72], EEG data was collected during resting state and an improvisation task using an EMOTIV EPOC + 14-channel wireless headset, sampled at 128 Hz. For each participant, relative power spectral density within the theta (4–8 Hz) and alpha (8–13 Hz) bands from the frontal and parietal regions was extracted across both conditions and averaged accordingly [72]. EEG data preprocessing was conducted using Brain Electrical Source Analysis software (BESA Research, version 7.1) [72].

Further, significant variation existed in the acquisition and processing of structural MRI data across the five studies [67–71]. Four studies acquired and analysed T1 data. Out of these four studies, three utilised voxel-based morphometry (VBM) [68, 70, 71], and the last one used FreeSurfer [67] to obtain GM volumes from their T1-weighted images. Further, two studies acquired and analysed DWI data. Out of these two studies, one ran a fixel-based analysis (FBA) using MRtrix3 [69] to calculate FBA metrics such as fibre density, fibre-bundle cross-section, and fibre density and cross-section, while the other study focused on diffusion tensor imaging (DTI) using FSL FDT (FMRIB's Diffusion Toolbox—diffusion tensor) to obtain GM and WM fractional anisotropy and mean diffusivity [67]. The significant differences in analysis techniques make it difficult to compare the results across these structural MRI studies. Furthermore, the brain regions reported in the included studies also differed considerably. For T1-weighted images, the most frequently analysed brain regions were total GM volumes [67, 70], hippocampal volumes [67, 68] and cerebellum volumes [68, 70]. For DWI, there was no overlap in the brain regions that were analysed, and it was therefore not possible to draw comparisons between them [67, 69] (see Table 1 and Table 3 for details). No studies used functional MRI or molecular (e.g. magnetic resonance spectroscopy) methods.

Regarding our secondary aim, five of the six studies included in this review analysed the association between brain changes and cognition, with memory being the most frequently reported cognitive outcome [68–70].

### Risk of bias assessment

Findings from the risk-of-bias assessment are summarised in Table 2. Only one study [67] was assessed as having an overall low risk of bias. A second study was rated as having an overall medium risk of bias [72]. The remaining four studies [68–71] were evaluated as having an overall high risk of bias with mixed results across the five domains. Some of the most common reasons for high risk of bias were the lack of description of allocation concealment and blinding, number of dropouts, and missing intention-to-treat-analysis.

**Table 2:**
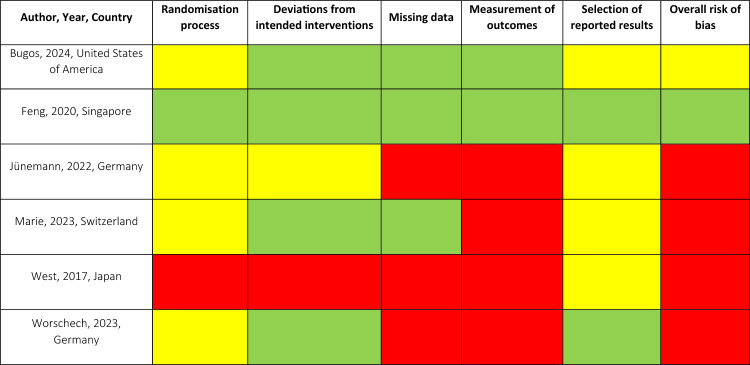
Risk of Bias Assessment

Green = Low risk of bias, Yellow = Medium risk of bias, Red = High risk of bias

### Effects of music-making intervention on neurobiological changes in cognitively intact older adults

Table 3 presents the associations between music-making interventions and neurobiological changes in older adults without objective cognitive impairment. The only study investigating the effects of piano training on neuronal oscillatory activity (EEG) found a greater frontal theta power in the piano group (n = 20) compared to the control group (n = 20) during their improvisation continuation task only at post-testing (p = 0.024) [72]. No other significant differences were observed for other EEG markers [72]. Among the four studies that examined GM volumes, only one reported a positive effect of piano training intervention on GM volumes. West et al. [68] found significant increases in GM volumes in the right cerebellum (t = 6.79, p < 0.05) and the left (t = 5.79, p < 0.05) and right (t = 5.67, p < 0.05) dorsolateral prefrontal cortex (DLPFC) in healthy older adults (n = 12) after six months of piano training when compared to the passive control group (n = 13). Additionally, significant reductions in GM volumes in the right hippocampus (t = 6.21, p < 0.05) were observed in the passive control group when compared with the piano training group [68]. No significant difference in GM volumes was found between the piano and active control (i.e. video game; n = 13) groups after six months [68].

**Table 3:** Effect of Music-Making Interventions on Neurobiological Changes in Adults Without Cognitive Impairment

First author, (publication year)	Brain Regions-of-interest (ROIs)	Modality (Processing technique)	Cognitive status (HOA/SCI/MCI)	Longitudinal differences^1^ between groups	Additional brain findings	Cognition results related to brain changes
Bugos (2024)	Frontal and parietal lobes	EEG resting-state and improvisation task (BESA)	HOA	At post-testing, the jazz piano group showed significantly greater frontal theta power during improvitation than the control group (p = 0.024) after 12-weeks	n/a	A negative correlation between errors committed on the Stroop task and PASAT scores as theta power increased
Feng (2020)	Global GM and WM Total brain volume, total GM volume, total WM volume, total ventricular volume, and hippocampal volume	DWI (FSL FDT- DTI) and T1 (FreeSurfer)	SCI	Non-significant	n/a	No significant associations between brain metrics and cognition
Jünemann (2022)*	Corpus callosum, fornix, acoustic radiation, corticospinal tract, and arcuate fasciculus	DWI (Fixel-Based Analysis)	HOA	The active control group experienced significant declines in fibre density of the fornix after 6 months of intervention (t[61] = 4.584, p < 0.001, d = 0.582, CI = 0.033, 0.013)	n/a	A positive correlation between the fornix fiber density and RAVLT-Delayed recall changes when groups were combined
Marie (2023)*	Total GM volume, primary auditory area, cerebellum, caudate nucleus and Rolandic operculum	T1 (VBM)	HOA	GM volumes in the right TE1.0 of the primary auditory cortex significantly declined in the control group after 6 months (F [1;126] = 5.5, p = 0.026)	GM volume increase at whole-brain level in the left caudate nucleus (t = 5.66; p = 0.003), right rolandic operculum (t = 5.49; p = 0.007) and right (t = 6.69; p < 0.001) and left (t = 5.67; p = 0.003) inferior cerebellum when merging both groups	Increased GM volumes in the bilateral cerebellum and training time lead to greater improvements in working memory when both groups were combined
West (2017)	Hippocampus, cerebellum, and DLPFC	T1 (VBM)	HOA	Significant reduction of GM volumes in the right hippocampus in the passive control group when compared to the music-making intervention (t = 6.21; p < 0.05) The music-making intervention group revealed increased volumes in the cerebellum (t = 6.79; p < 0.05) and left (t = 5.79; p < 0.05) and right (t = 5.67; p < 0.05) DLPFC when compared to the passive control group	n/a	Short-term memory performance improvement was associated with greater left hippocampal GM volumes across all participants
Worschech (2023)*	Primary motor cortex, thalamus and putamen	T1 (VBM)	HOA	Non-significant	Decreased GM volumes were observed in the bilateral putamen (right: -0.25, CRIs = [0.49, 0.01], p = 0.039; left: 0.23, CRIs = [0.46, 0.01], p = 0.065) and M1 (left: -0.37, CRIs = [0.62, 0.12], p = 0.004; right: -0.32, CRIs = [0.56, 0.07], p = 0.012) in the piano group (within group analysis) Decreased GM volumes were observed in the bilateral M1 (left and right: 0.39, CRIs = [0.63, 0.14], p = 0.002) and left putamen (0.36, CRIs = [0.61, 0.12], p = 0.002)) in the control (within group analysis)	n/a

*SCI* Subjective cognitive impairment, *HOA* Healthy older adults, *EEG* electroencephalography, *BESA* Brain Electrical Source Analysis software, *DWI* Diffusion-weighted imaging, *DTI* Diffusion tensor imaging, *GM* Grey matter, *WM* White matter, *RAVLT* Rey Auditory Verbal Learning Test, *DLPFC* Dorsolateral prefrontal cortex, *VBM* Whole-brain voxel-based morphometry, *FSL* FMRIB Software Library, *FD* FMRIB’s Diffusion Toolbox, *M1* Primary motor cortex, *n/a* non-applicable

*These studies used data from the same longitudinal study conducted in Hannover (Germany) and Geneva (Switzerland)

^1^Difference between follow-up and baseline

In contrast, Marie et al.'s study [70] found that the volumes of one subfield area (right TE1.0) in the primary auditory cortex remained stable in the piano group (n = 66), while there was a significant decrease in GM volumes in the TE1.0 area in the control group (n = 66) over six months of intervention (t = 5.5, p = 0.026). However, the specific post-hoc test used in this study was chosen based on the biggest difference in means after a significant three-way interaction effect with more than two levels for the factor encoding brain region. The validity of this procedure has been debated [74–76]. Lastly, Worschech et al. [71] and Feng et al. [67] did not find any significant difference in global and total brain, primary motor cortex (M1), hippocampus, thalamus and putamen GM volumes between their music-making interventions (i.e. piano (n = 68) and choral singing (n = 46) training) and their active control groups after one year and two years of intervention, respectively.

None of the studies that used DWI [67, 69] showed a significant positive effect of the music-making intervention on brain metrics. However, one study [69] calculated the difference scores between 6 months and baseline and compared those between the two groups. The study found a significant difference in fibre density in the fornix (t = 4.58, p < 0.001) between the intervention group (n = 59) and the active control group (n = 62). To investigate further, a one-sample t-test for both groups was conducted [69]. The results showed that the active control group experienced a decline in fibre density over six months, while the intervention group did not reveal any fibre density changes over time [69].

### Further MRI findings

Worschech et al. [71] performed an additional within-group analysis and significant decreases in GM volumes in the bilateral putamen (right: − 0.25, CRIs = [0.49, 0.01], p = 0.039; left: − 0.23, CRIs = [0.46, 0.01], p = 0.065) and M1 (left: − 0.37, CRIs = [0.62, 0.12], p = 0.004; right: − 0.32, CRIs = [0.56, 0.07], p = 0.012) in the piano training group (n = 68) during the second semester of the intervention. Additionally, significant decreases in GM volumes were observed in the bilateral M1 (left and right: − 0.39, CRIs = [0.63, 0.14], p = 0.002) and the left putamen (− 0.36, CRIs = [0.61, 0.12], p = 0.002) in the active control group during the first six months of the intervention [71]. Overall, both groups showed reductions in GM volumes at 12 months. However, it is noteworthy that this decline was not significantly different between groups.

Further, Marie et al.'s study [70] did not identify a positive effect of their piano training intervention on the GM volumes of the cerebellum, caudate nucleus, and rolandic operculum. Nonetheless, the researchers utilised a data-driven approach by combining both groups (i.e. piano training and music listening). This merger yielded significant increases in GM volume at the whole-brain level in the left caudate nucleus (t = 5.66, p = 0.003), right rolandic operculum (t = 5.49, p = 0.007), and right (t = 6.69, p < 0.001) and left (t = 5.67, p = 0.003) inferior cerebellum [70].

### Association between neurobiological changes and cognitive measures

Only one study reported a significant association between neurobiological changes and cognitive improvements following a music-making intervention [72] (see Table 3 for details). Bugos et al. [72] conducted a bivariate correlation analysis to examine the relationship between inhibition performance, processing speed, and frontal theta power during their improvisation continuation task. At post-testing, they found a significant negative correlation between errors committed on the Stroop task and Paced Auditory Serial Addition Task (PASAT) scores as theta power increased (r = − 0.446, p = 0.014) [72]. Further, as an exploratory analysis, Jünemann et al. [69] found a weak positive correlation (Spearman’s r = 0.237, p = 0.0097) between changes in fornix microstructure and episodic memory when both music intervention groups were combined (i.e. piano training and music listening). Similarly, Marie et al. [70] found a significant interaction effect between training intensity and bilateral cerebellar GM volumes, indicating that increased training time and GM volumes in the bilateral cerebellum lead to greater improvements in working memory when both groups, piano training and music listening, were combined (F (1: 100) = 9.9, p = 0.013). One study [71] did not examine the association between their cognitive data and neurobiological changes.

## Discussion

The purpose of this systematic review was to assess the effect of music-making interventions on neurobiological changes in older adults with and without MCI, via examination of neuroimaging markers. Overall, the results showed that the studies were of limited quality. Five of the six studies examined structural MRI endpoints but utilised varied outcome measures and analytical techniques from pre-specified regions of interest [67–71] to data-driven approaches [70, 71]. Only one study assessed EEG data [72]. Thus, results across these studies are difficult to synthesise.

Notably, our search did not reveal any studies in older people with MCI that met our inclusion criteria. Altogether, six studies with a total of 555 participants comprising healthy older adults (N = 462) and those with SCD (N = 93) were included in this review. Sample sizes varied from 33 to 136. Of these, only two reported a positive effect of a piano training intervention on brain metrics [68, 72]. However, direct comparisons between these two studies are challenging due to the differences in neuroimaging modalities, intervention duration, and outcome measures. The study by Bugos et al. [72] reported a significant increase in frontal theta power in the piano group compared to the control group during their improvisation continuation task after 2-weeks of intervention. This enhancement in frontal theta power may indicate improvements in cognitive control and attention, as theta oscillations in the frontal cortex are associated with executive functions such as working memory and attentional processes [77, 78]. Nevertheless, further studies with larger sample sizes and active control groups are needed to better understand the relationship between frontal theta power, executive functioning, and piano training. Further, the study by West et al. [68] revealed increased GM volumes in the DLPFC and cerebellum. In addition to the small sample size of 20 subjects, the original manuscript noted that the control arm of this study experienced high attrition rates, resulting in the inclusion of new participants without undergoing randomisation. Hence, the calibre of this study may be compromised, making it difficult to draw definitive conclusions.

Furthermore, three studies suggested a stabilising effect of piano training interventions on structural brain markers, thereby supporting the notion that music interventions could mitigate cognitive decline. The control groups exhibited significant declines in fibre density in the fornix [69], as well as GM volume in the right TE1.0 in the primary auditory cortex [70] and right hippocampus [68], while the intervention groups did not experience any significant brain changes in those brain regions. These results suggest that piano training may slow down age-related brain atrophy. However, it is noted that two out of the three studies [69, 70] were conducted utilising data gathered from the same participants of an international multisite study of 155 older people. This overlap in participants introduces a dependency that could confound the results. Specifically, this can result in an overestimation of the intervention's effectiveness and limit the generalizability of the findings to the broader population. Therefore, further research is necessary to replicate these results with independent cohorts to confirm the findings.

Two out of the six studies investigated neurobiological outcomes for all participants, pooled across their music-making and control intervention groups [70, 71]. Importantly, the control condition received an active intervention that included a musical element (i.e. music listening/musical culture lessons). Interestingly, these studies found significant decreases in GM volumes in the putamen and M1, as well as increased GM volumes in the cerebellum, caudate nucleus, and rolandic operculum in both groups (i.e. piano training and music listening) after one year [71] or 6-months [70] of intervention. This finding suggests that the impact of these interventions on GM volumes may be attributed to the musical aspect of both interventions rather than a distinct feature of the piano intervention. However, the lack of a passive or non-music-related control condition in these particular studies limits this interpretation. Additionally, for Worschech et al. study [71], the music listening control group had a higher dropout rate (n = 18) in comparison to the piano training intervention group (n = 4). Thus, caution must be exercised when interpreting these results considering the significant likelihood of bias.

There is limited evidence to guide the prescription of music-making interventions to promote neurobiological changes in older adults at risk for dementia. While both interventions used in the included studies (i.e. piano training and choral singing) integrate musical and social components, there are important differences between them. For instance, choral singing requires the acquisition of breathing and posture techniques and focuses on verbal memory [79]. Piano training, on the other hand, involves learning how to read musical scores, refining fine motor skills, and attaining metric accuracy [80]. It also demands memorisation of sound and movement patterns [80]. For this systematic review, five of the six studies we analysed utilised piano training [68–71], while only one used a choral singing intervention [67]. Interestingly, the study that used a choral singing intervention (n = 47) for a two-year period did not yield any significant results on any pre-specified MRI-derived brain marker [67]. This may suggest that the components of piano training may have more robust neurobiological effects than a choral singing intervention. However, a key limitation of Feng et al. [67] study is that it did not specify whether participants had prior musical experience, making it unclear whether pre-existing musicianship influenced the results. The lack of significant findings may be partially attributed to a failure to control for musicianship within the sample. Furthermore, four of the five studies that utilised piano training were assessed as having a high risk of bias, and three out of the four studies were based on data from the same international multisite study. Consequently, these findings should be interpreted with caution, and further research with independent cohorts and rigorous methodologies is essential to validate the different effects of piano training and choral singing on the brain.

Additionally, cognition was examined in the five studies that implemented piano training as an intervention [68–72]. In contrast to the approach by Feng et al. [67], these studies utilised a global cognitive assessment to screen for dementia and did not employ a comprehensive neuropsychological battery to assess for cognitive decline. Global cognitive tests provide a broad overview of cognitive function but do not capture specific cognitive domains, potentially overlooking subtle impairments in areas such as memory, learning and executive function [81, 82]. Without a detailed assessment, individuals with MCI might be misclassified as cognitively healthy. This misclassification can introduce variability in the baseline cognitive status of participants, leading to confounding effects in the imaging results. Therefore, it is crucial to conduct further research involving these two types of music interventions with well characterised populations to gain a deeper understanding of their respective effects on structural and functional brain markers.

Regarding our second aim, although prior evidence suggests that music-making interventions can have positive effects on episodic memory and executive functioning in older adults at risk for dementia [57], this systematic review found limited evidence supporting neurobiological changes underlying these cognitive improvements. Of the six included studies, only one demonstrated an association between neurobiological changes and cognitive improvement in the intervention group. Bugos et al. [72] reported a significant positive correlation between executive functioning improvements and greater frontal theta power, suggesting that neural oscillatory activity—particularly frontal theta power during a musical improvisation task—may be a more sensitive and dynamic marker of neurobiological changes than structural brain alterations, especially following a short piano training period (i.e., two weeks). These findings underscore the potential of functional neuroimaging techniques as early biomarkers for detecting cognitive changes, often preceding observable structural changes. However, further research directly comparing both structural and functional neuroimaging data is crucial to elucidate the temporal dynamics of music-induced brain plasticity.

Interestingly, although the other five studies did not find a significant association between neurobiological changes and cognition in the intervention group, two studies [69, 70] observed a positive effect between change in structural MRI markers and memory performance when combining the intervention and active control groups (i.e. music listening/musical culture lessons). This suggests that the effect of structural brain changes on memory performance may not be specific to music-making interventions (e.g. piano training or choral singing). Rather, other factors, such as social engagement levels [83] may also play a role. However, the presence of significant results when both groups are combined may also be a consequence of bias associated with the study design [84], or increased statistical power in a larger, pooled sample. Hence, further research is necessary to understand the unique effects of music-making interventions on neurobiological changes and cognition in this population.

Several limitations were detected in the five studies that were part of this review. First, as noted, the majority (4/6) had a high risk of bias. The predominant issue was the absence of an adequate explanation regarding the blinding procedure. Given the inherent nature of music-making interventions, concealing the treatment allocation from the participants is not possible. Nonetheless, most of these studies failed to indicate whether the outcome assessors were blinded, which may have impacted the analysis of results due to knowledge of the intervention. Three studies had high attrition rates and missing data, which could have impacted the validity of their results. Two studies [68, 72] failed to pre-register their clinical trial and did not disclose conducting a power analysis to guide sample size determination and study design. Second, as noted above, the five studies [68–72] that used piano playing as an intervention relied solely on global cognitive assessments to recruit healthy older adults rather than employing a comprehensive neuropsychological battery. This limitation may have led to the misclassification of individuals with MCI, introducing variability in the baseline cognitive status of participants and potentially confounding the imaging results.

Further, the six studies used a hypothesis-driven approach to specify their neuroimaging outcomes. However, due to limited literature on the relationship between music-making intervention and neuroimaging markers, the neuroimaging outcomes of these RCTs are primarily exploratory. Hence, there was significant variability in the brain metrics used to assess the effect of music-making interventions on neurobiological changes, even within the limited studies reviewed. This is a key consideration in interpreting the results since findings about structural and functional brain changes may be contingent upon the specific neuroimaging modality utilised and the particular region of the brain that has been examined. To enhance comparability across studies, future research should prioritise standardising ROI selection by focusing on brain regions previously associated with both musicians and dementia populations. These ROIs should also be linked to established dementia markers (e.g. cognitive performance, APOE4 status) to better clarify their role in neuroprotection and disease progression. Finally, of the six studies included in this review, five utilised structural MRI to examine neuroplasticity changes, while only one study employed a functional method (i.e. EEG). Hence, there is a significant gap in the literature regarding the functional neural mechanisms underlying music-making interventions in older adults. Future research should prioritise functional neuroimaging approaches to capture short-term neuroplastic adaptations to music-making interventions. Modalities such as EEG and functional near-infrared spectroscopy (fNIRS) offer cost-effective, accessible, and flexible methods to investigate real-time functional changes with reduced participant burden and attrition. EEG/fNIRS could provide in vivo insights into the neural correlates of music production, facilitating a deeper understanding of how engagement in music training influences dynamic brain activity.

Despite the limited number and varied quality of available RCTs, we proceeded with this systematic review to critically assess the strongest evidence on the effects of music-making interventions on brain plasticity in older adults. As RCTs remain the gold standard for evaluating causal relationships, focusing exclusively on these studies provided a rigorous appraisal of the current literature. Therefore, this review holds particular value for several reasons. Firstly, it is important to highlight the significant gap in the existing body of research. While it is commonly believed that music promotes brain plasticity, there is currently no definitive proof of this effect in older adults with and without MCI. By continuing with this systematic review, we aimed to underscore the lack of robust evidence in this area and the need for more high-quality studies. Secondly, this review consolidates the existing evidence, providing a clearer picture of what has been studied so far and identifying consistent findings and discrepancies. This consolidation is essential for guiding future research directions and ensuring that subsequent studies build on a solid foundation of existing knowledge. Lastly, by identifying the methodological limitations and variations in the current studies, we can offer recommendations for improving the design and execution of future research. This includes advocating for larger sample sizes, more rigorous randomisation processes, and standardised outcome measures, which are crucial for obtaining more reliable and generalisable results.

Moreover, we acknowledge that an important limitation of this systematic review is that three of the six included studies relied on information gathered from the same participants in a single international study conducted across multiple sites. Given the overall scarcity of studies investigating neurobiological outcomes following music-making interventions, we decided to proceed with the review; however, we note that this requires cautious interpretation and limits generalisation of these results.

Unfortunately, despite conducting a thorough systematic review of the available literature on neurobiological changes associated with music-making interventions in older adults, we could not locate any studies involving individuals with MCI. Hence, we have identified a significant gap in the existing literature which needs to be addressed in the context of international recommendations for early non-pharmacological intervention to reduce the risk of cognitive decline. This is especially important given that those with MCI are at higher risk for conversion to dementia [7]. Common public health messaging and health professional advice suggest taking up challenging hobbies or learning new skills, such as instrument training or singing, to promote neuroplasticity in aging. However, the apparent lack of empirical evidence to support such suggestions with respect to musical engagement undermines this messaging. As such, further research is required to ascertain the underlying neurobiological effects associated with music-making in older adults, and particularly in those with MCI. Studies should also incorporate blood-based biomarkers (e.g. tau-217, NfL) to assess the effects on other aspects of disease pathology and to examine whether certain ApoE4 subgroups derive less benefit. This approach has been found useful in other intervention studies, including recent monoclonal antibody (Mab) trials [85]. Such evidence will enable appropriate design of structured or group-based interventions involving music-making that may be used to facilitate brain plasticity and ultimately improve or maintain cognitive functioning in this population.

## Conclusion

To our knowledge, this systematic review is the first to evaluate the neurobiological effects of music-making interventions in healthy older adults or those with mild levels of cognitive impairment. Aside from the small number of studies, there were methodological limitations and heterogeneity in interventions and neuroimaging outcomes precluding solid conclusions in either cognitively intact older adults and MCI. Additionally, three studies included the same participants, introducing a dependency that could confound the results and limit their generality. Further, well designed RCTs alongside common or standardised neuroimaging and other neurobiological markers of neuroplasticity and/or cognitive decline are required in order to ascertain the use of music-making interventions or training as a cognitive strategy in older adults. Given the intrinsic and broad interest in music, if an empirical evidence base can be determined for its capacity to promote neuroplasticity, there is clear potential for exploring how such interventions may be implemented within community and/or public health settings.

## Supplementary Information

Below is the link to the electronic supplementary material.

Supplementary file1 (DOCX 24 KB)

## Data Availability

No datasets were generated or analysed during the current study.
