# Supplementary material for: Neurobiological effects of music-making interventions for older adults: a systematic review
Source: Aging Clin Exp Res. 2025 Apr 2;37(1):113. doi: 10.1007/s40520-025-03006-7 (PMC11965234; doi:10.1007/s40520-025-03006-7)
Supplement: Supplementary file 1 — Supplementary file1 (DOCX 24 KB) [file 40520_2025_3006_MOESM1_ESM.docx]

**Supplementary Material**

**Search strategy**

**Database:** PsycInfo, Medline and Scopus

**Limited to English:** No

**Date Range:** No date limits

**Publication types**: All types

| 1 | “Geriatric patients” OR “healthy ag*ng” OR ag*ng OR elderly OR “older adults” OR “older people” OR “older participants” OR age-related OR geriatric* |
| --- | --- |
| 2 | “Cognitive dysfunction” OR “mild cognitive impairment” OR “cognitive ability” OR “cognitive ag*ng” OR “cognitive impairment” OR “mild cognitive deficit” OR “cognitive decline” OR “minimal cognitive impairment” OR “prodromal alzheimer*” OR at-risk OR pre-Alzheimer* OR prealzheimer* OR “preclinical Alzheimer*” OR “pre-clinical Alzheimer*” OR pre-dementia OR predementia OR “preclinical dementia” OR “prodromal dementia” |
| 3 | 1 OR 2 |
| 4 | “Musical ability” OR “musical instruments” OR “music making” OR music-making OR music* OR “music intervention” |
| 5 | Choral OR choir OR chorus OR vocali*ation OR voice OR singing OR singer* OR orchestra* |
| 6 | Piano OR pianist* OR percussion OR drum OR guitar OR flute |
| 7 | 4 OR 5 OR 6 |
| 8 | Neuroanatom* OR neuroimag* OR anatom* OR cerebral OR brain OR imaging OR neurobiological OR “neural correlate” OR neuroplasticity OR plasticity or “biological neural networks” |
| 9 | “Magnetic Resonance Imaging” OR “Diffusion Tensor Imaging” OR “Perfusion Imaging” OR “Diffusion Magnetic Resonance Imaging” OR Spectroscopy OR electroencephalography OR “computed tomography” OR CT OR x-ray OR electroencephalogram OR electroencephalogr* OR EEG OR Tomography OR “Positron emission tomography” OR PET OR “Single Photon Emission Computed Tomography” OR SPECT OR “magnetic resonance*” OR *spectroscopy OR fMRI OR MRI OR MRS OR angiography OR magnetoencephalography OR MEG |
| 10 | “White matter” OR “gr*y matter” OR cortical OR “brain volume*” OR “BOLD signal” OR blood-oxygen* OR functional OR structural OR perfusion OR “blood flow” OR morpho* OR volumetric OR “neural correlate*” OR metabolite* OR susceptibility-weighted* OR microbleed* OR diffusion* OR tract* OR vascular* OR subcortical OR connectivity OR “brain structure” OR “brain size” OR “cortical thickness” OR hippocamp* OR “frontal cortex” OR prefrontal OR “auditory cortex” OR “cerebral cortex” OR “temporal cortex” OR “parietal cortex” OR “basal ganglia” |
| 11 | 8 OR 9 OR 10 |
| 12 | “Randomi*ed controlled trial” OR intervention OR “clinical trials” OR “pilot study” OR RCT |
| 13 | 3 AND 7 AND 11 AND 12 |
